# Quasi-real-time range monitoring by in-beam PET: a case for ^15^O

**DOI:** 10.1038/s41598-023-45122-2

**Published:** 2023-11-01

**Authors:** S. Purushothaman, D. Kostyleva, P. Dendooven, E. Haettner, H. Geissel, C. Schuy, U. Weber, D. Boscolo, T. Dickel, C. Graeff, C. Hornung, E. Kazantseva, N. Kuzminchuk-Feuerstein, I. Mukha, S. Pietri, H. Roesch, Y. K. Tanaka, J. Zhao, M. Durante, K. Parodi, C. Scheidenberger

**Affiliations:** 1 GSI Helmholtzzentrum für Schwerionenforschung GmbH, Darmstadt, Germany; 2 Department of Radiation Oncology, Particle Therapy Research Center (PARTREC), University Medical Center Groningen, University of Groningen, Groningen, The Netherlands; 3 II. Physikalisches Institut, Justus-Liebig-Universität, Gießen, Germany; 4 Department of Electrical Engineering and Information Technology, Technische Universität Darmstadt, Darmstadt, Germany; 5 Institute for Nuclear Physics, Technische Universität Darmstadt, Darmstadt, Germany; 6 RIKEN Cluster for Pioneering Research, RIKEN, Wako, Japan; 7 School of Physics, Beihang University, Beijing, China; 8 Department of Condensed Matter Physics, Technische Universität Darmstadt, Darmstadt, Germany; 9 Department of Medical Physics, Faculty of Physics, Ludwig-Maximilians Universität München, Munich, Germany; 10 Helmholtz Forschungsakademie Hessen für FAIR (HFHF), Campus Gießen, Gießen, Germany

**Keywords:** Applied physics, Biological physics, Cancer therapy, Cancer therapy, Biophysics

## Abstract

A fast and reliable range monitoring method is required to take full advantage of the high linear energy transfer provided by therapeutic ion beams like carbon and oxygen while minimizing damage to healthy tissue due to range uncertainties. Quasi-real-time range monitoring using in-beam positron emission tomography (PET) with therapeutic beams of positron-emitters of carbon and oxygen is a promising approach. The number of implanted ions and the time required for an unambiguous range verification are decisive factors for choosing a candidate isotope. An experimental study was performed at the FRS fragment-separator of GSI Helmholtzzentrum für Schwerionenforschung GmbH, Germany, to investigate the evolution of positron annihilation activity profiles during the implantation of $^{14}$O and $^{15}$O ion beams in a PMMA phantom. The positron activity profile was imaged by a dual-panel version of a Siemens Biograph mCT PET scanner. Results from a similar experiment using ion beams of carbon positron-emitters $^{11}$C and $^{10}$C performed at the same experimental setup were used for comparison. Owing to their shorter half-lives, the number of implanted ions required for a precise positron annihilation activity peak determination is lower for $^{10}$C compared to $^{11}$C and likewise for $^{14}$O compared to $^{15}$O, but their lower production cross-sections make it difficult to produce them at therapeutically relevant intensities. With a similar production cross-section and a 10 times shorter half-life than $^{11}$C, $^{15}$O provides a faster conclusive positron annihilation activity peak position determination for a lower number of implanted ions compared to $^{11}$C. A figure of merit formulation was developed for the quantitative comparison of therapy-relevant positron-emitting beams in the context of quasi-real-time beam monitoring. In conclusion, this study demonstrates that among the positron emitters of carbon and oxygen, $^{15}$O is the most feasible candidate for quasi-real-time range monitoring by in-beam PET that can be produced at therapeutically relevant intensities. Additionally, this study demonstrated that the in-flight production and separation method can produce beams of therapeutic quality, in terms of purity, energy, and energy spread.

## Introduction

Proton therapy is currently the most widespread type of ion beam therapy. The rationale behind using ions heavier than protons for radiation therapy is the reduced lateral scattering with increasing ion mass and the higher relative biological effectiveness (RBE)^1^ in the tumor region. The facility for ions heavier than protons has a downside characterized by higher investment costs, typically ranging from 2 to 4 times more expensive and the cost per treatment of carbon ions is about 2–3 times higher than that of conventional therapy with X-rays^2^. Additionally, the heavy ions have the issue of unavoidable projectile fragmentation, which leads to an undesirable dose tail distal to the target. Carbon has been identified as an excellent compromise ion due to its favorable characteristics. It exhibits the best ratio of biologically effective dose in the tumor compared to the entrance channel for numerous indications. Consequently, carbon is presently the most widely utilized ion at all light ion beam therapy centers. Other light ions are also being considered, with oxygen being one of them^3–7^. The main rationale for using ions heavier than $^{12}$C is to further increase the particle’s linear energy transfer (LET), which can effectively target hypoxic tumors. Oxygen, despite having a linear energy transfer (LET) that is 80% higher than carbon, is often deemed too aggressive for most indications. However, it holds potential for application in multi-ion painting particle therapy. In this therapy approach, where multiple ion species are used to target different regions within a tumor, the higher LET of oxygen could potentially enhance the therapeutic outcomes^7–9^.

The dose and/or LET gradients induced in the volumes irradiated by light ions raise the need for precise beam range monitoring to ensure the compromise between the uniform target coverage and the sparing of the surrounding healthy tissue and critical structures. With no direct feedback on dose conformity during the treatment, ion beam therapy relies solely on the accuracy of treatment planning procedures and systems. Additionally, patient anatomical changes may occur during treatment. To address this, margins are typically added to the treated region, which unfortunately causes unnecessary dose deposition to healthy tissue, ultimately leading to increased toxicity^10^. Positron emission tomography (PET) is one of the most commonly employed methods to monitor dose delivery in ion beam therapy^11^. Here the positron-emitters are produced through fragmentation processes of the atomic nuclei in the beam and the tissue, and Monte Carlo codes are typically used to correlate the PET data with the actual dose profile. However, this process is typically performed as a post-irradiation procedure to validate treatment plans. In contrast to protons, the PET image of a $^{12}$C ion beam has an identifiable peak produced by the positron-emitting projectile fragments, but its mismatch with the primary beam range^12, 13^, the low statistics at typical fraction doses and the long half-life of the most abundantly produced positron-emitting projectile fragment ($^{11}$C) complicate fast in-beam PET range monitoring in clinical settings and limits the PET-based range verification accuracy to approximately 2–5 mm^14, 15^. The same challenges also apply to an $^{16}$O ion beam^13, 16, 17^.

Several ideas for a feedback system are being explored to mitigate this problem. Among these, image-guided ion beam therapy with positron-emitting radioactive beams in combination with PET is one of the most promising ones. The general idea behind utilizing positron emitters as therapy beams is to achieve sub-millimeter precision for range verification through PET. This idea was pursued early on during the pilot project on heavy ion beam therapy at the Lawrence Berkeley Laboratory (LBNL)^18^. The merits of ion beam therapy using positron emitters have been extensively reviewed by multiple authors^19, 20^. The Heavy Ion Medical Accelerator in Chiba (HIMAC), Japan, investigated the feasibility of using secondary beams of  $^{11}$C and $^{15}$O positron emitters, produced by projectile fragmentation, for both therapy and in-beam PET range verification^21–23^.

To fully realize the benefits of positron-emitting therapy beams, it is crucial to have quasi-real-time feedback on any potential deviations from the treatment plan with minimal exposure to healthy tissue. Since the quasi-real-time approach requires in-beam PET, it avoids the relocation of the patient, which pose a challenge to accurate patient positioning. Additionally, since the patient relocation process itself typically takes at least five minutes, the imaging quality can be negatively affected by biological washout^24–27^. It must be noted that a fast image reconstruction algorithm and an adequate computational infrastructure are equally important in exploiting the benefits of quasi-real-time range verification offered by short-lived positron-emitting therapy beams. In this sense, relying on 2D feedback is a more pragmatic approach.

The BARB (Biomedical Applications of Radioactive Beams) project at GSI, aims at pre-clinical validation of in vivo beam visualization in heavy ion beam therapy with positron emitting carbon and oxygen isotopes^28–31^. The project also conducts related basic studies. As a part of this project, experiments were performed with the fragment separator FRS studying the evolution of the PET image during irradiation with positron emitters of carbon ($^{10}$C and $^{11}$C) and oxygen ($^{14}$O and $^{15}$O). The properties of these isotopes are shown in Table 1.

**Table 1:** The characteristics of oxygen and carbon isotopes relevant for this study.

Isotope	Half-life (s)	Prompt–$\gamma$ emission	$\beta ^{+}$-emission (MeV) end-point energy	RMS effective (mm) positron range in water
$^{15}$O	122.24 (16)	None	1.732	1
$^{14}$O	70.606 (18)	2.313 MeV at 99.4% of decays	1.808	1.1
$^{11}$C	1220.84 (97)	None	0.96	0.4
$^{10}$C	19.310 (4)	0.718 MeV at 100% of decays	1.90	1.1

The root mean square (RMS) effective range is given to represent the blurring caused by the positron range distribution^32^.

The positron emitters were produced and separated in-flight by the FRS and implanted in a polymethyl methacrylate (PMMA) phantom for PET imaging studies using an in-beam dual panel PET scanner. The primary goal of the experiments was to establish a relationship between the minimum amount of detected decays required to achieve a range verification precision better than the typical systematic uncertainty of the patient positioning systems. The results from the carbon experiment, which provide a detailed description of the experimental setup and methodology, have already been published^30^. This article reports on an experiment with positron emitters of oxygen, $^{14}$O and $^{15}$O, and the results are compared to the data obtained for $^{10}$C and $^{11}$C within the context of quasi-real-time in-beam range monitoring^30^.

## Materials and methods

### Positron emitting beams

The FRS at GSI, where this experiment was conducted, is an in-flight secondary-beam facility^33^. Secondary beams of positron emitters of oxygen were produced by projectile fragmentation of the $^{16}$O ion beams accelerated by the SIS18 synchrotron and impinging on an 8 g/cm$^2$ thick beryllium production target at the entrance of the FRS. Two different primary beam energies were used: 370 MeV/u (low-energy run) and 465 MeV/u (high-energy run). The secondary beams of interest were separated in-flight using the FRS with the well-established $B\rho$-$\Delta E$-$B\rho$ technique^34^. The FRS was operated in its standard ion-optical mode, characterized by being overall-achromatic with an acceptance of 20 $\pi$ mm mrad and momentum spread $\Delta p/p$ of 2$\%$ (FWHM). The angle of the wedge-shaped degrader was chosen such that the overall achromatism was preserved. The thickness of the wedge degrader was adjusted to have the same mean range during the implantation of different isotopes in an individual run. The definition of the “mean range” used in the following is the depth at which half of the particles are stopped. The estimated water equivalent mean range of low- and high-energy runs are 53 and 124 mm, respectively, according to the calculations with ATIMA 1.2 code using LISE++ program^35^. These values are chosen to represent typical values used in heavy ion beam therapy. Prior to irradiating the phantoms at high intensity for imaging purposes, the purity of the beam was evaluated through event-by-event particle identification at low intensity. For more details on the beam properties, see Table 2. During the imaging runs, a dedicated gas-filled parallel-plate ionization chamber (IC)^36^ was used to record the beam intensity.

### Imaging

**Figure 1:**
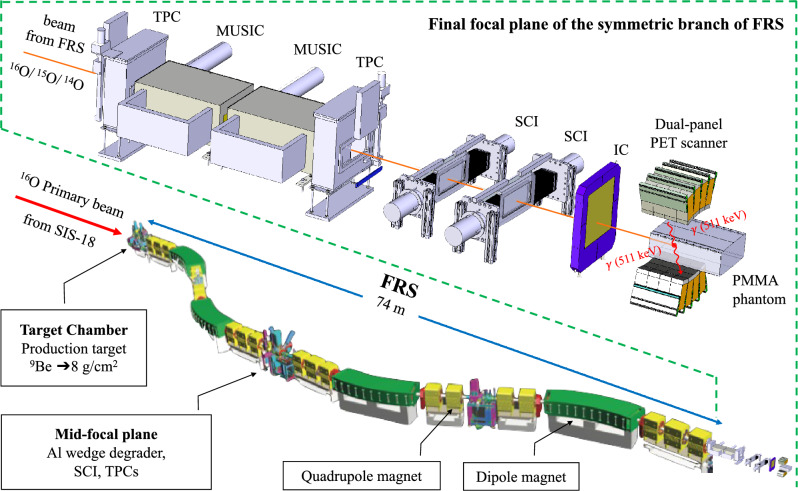
Schematic view of the FRS and a detailed view (inset in dashed line) of the experimental setup at the final focal plane of the symmetric branch of the FRS. The standard detectors of the FRS used for in-flight particle identification are, (i) plastic scintillators (SCI) for the time of flight determination (TOF); (ii) ionization chambers (MUSIC) for the energy deposition measurements to deduce the atomic number; and (iii) time projection chambers (TPC) for position measurements to deduce the magnetic rigidity ($B\rho$). The intensity of the beam was measured by a large area parallel plate ionization chamber (IC)^36^. The beam was implanted into the PMMA phantom placed in between the top and bottom panels of the PET scanner.

Isotopically pure beams of $^{14}$O, $^{15}$O and $^{16}$O (see Table 2) were delivered to the PET imaging setup at the final focal plane of the symmetric branch of the FRS (see Fig. 1). Ions were implanted in a homogeneous polymethyl methacrylate (PMMA) phantom for PET imaging studies using One-sixth of a modified version of a Siemens Biograph mCT clinical scanner, configured as a dual-panel scanner^37^. Each panel is composed of a 4$\times$4 array of Siemens Biograph mCT block detectors that cover a total area of 225$\times$220 mm$^2$. Each block detector consists of a 13$\times$13 array of 4$\times$4$\times$20 mm$^3$ LSO (lutetium oxyorthosilicate doped with cerium) scintillation crystals read out by a 2$\times$2 array of PhotoMultiplier Tubes (PMTs). The panels were placed 35 cm apart with the positioning structure for the PMMA phantom in between. The panels are curved around the beam axis with a radius of curvature of 42.1 cm. The geometrical efficiency of the scanner is estimated to be about 32% for a central point source^38^. The PMMA phantom has a size of 120$\times$250$\times$350 mm$^3$ and was placed in-between the two PET scanner panels with its long side in the beam direction and its short side in the vertical direction, see Fig. 1. After each measurement, the phantom was exchanged in order to avoid the influence of the previous activation. A system with sliding rails and positioning pins was employed to allow easy exchange and precise positioning of the phantom. Positioning of the phantom relative to the scanner’s field of view (FoV) along the beam axis was possible in steps of 35 mm with a precision ±0.25 mm. During the irradiation, the phantom positions were chosen such that the implantation depth of the beam was as close to the center of the FoV of the scanner as possible. The ion beam range in the phantom was calculated prior to the experiment with the ATIMA 1.2 code with the LISE++ program^35^.

**Table 2:** The relevant properties of the oxygen isotope beams for the two energy runs performed in this study.

Isotope	Beam energy [MeV/u]	Beam purity [%]	Momentum spread $\Delta p/p$ [%]	Cycle time structure		Average beam intensity	
				Beam ON [s]	Beam OFF [s]	Overall [ions/s]	Beam-ON [ions/pulse]
**High-energy run**							
$^{16}$O	295	99.6	0.3(1)	1	1.3	2.6$\times$10$^{7}$	6.0$\times$10$^{7}$
$^{15}$O	307	99.5	0.7(1)	1	1.5	4.3$\times$10$^{6}$	1.1$\times$10$^{7}$
$^{14}$O	320	95.8	0.7(1)	1	1.5	4.3$\times$10$^{5}$	1.1$\times$10$^{6}$
**Low-energy run**							
$^{16}$O	179	96.9	0.3(1)	1	1.3	8.2$\times$10$^{7}$	1.9$\times$10$^{8}$
$^{15}$O	185	99.6	0.8(1)	1	1.3	1.7$\times$10$^{6}$	4.0$\times$10$^{6}$
$^{14}$O	192	96.9	0.7(1)	1	1.3	8.3$\times$10$^{4}$	1.9$\times$10$^{5}$

For $^{16}$O implantation, the unreacted $^{16}$O that passed through the target was used instead of the direct primary beam from SIS18 to keep a similar material budget (production target and degrader) for all three isotopes. It is important to note that the figure legends and captions provide average overall intensities, which are calculated as the total number of implanted ions divided by the total beam-ON time plus the total beam-OFF time. Additionally, the average beam-ON intensities, which are obtained by dividing the average overall intensities by the duty cycle, are also provided for completeness.

#### Positron activity profiles and time structure of the implanted beams

Secondary beams produced by the FRS are inherently pulsed since the driver accelerator is a synchrotron. The nominal extraction time was chosen to be 1.0 s followed by a 1.3 or 1.5 s beam-off time, during which the next ion bunch was accelerated. A beam cycle is defined as a time period comprising both beam-on and beam-off times. The beam cycle time for high energy runs of $^{14}$O and $^{15}$O was 2.5 s, while for all other cases, it was 2.3 s, see Table 2.

**Figure 2:**
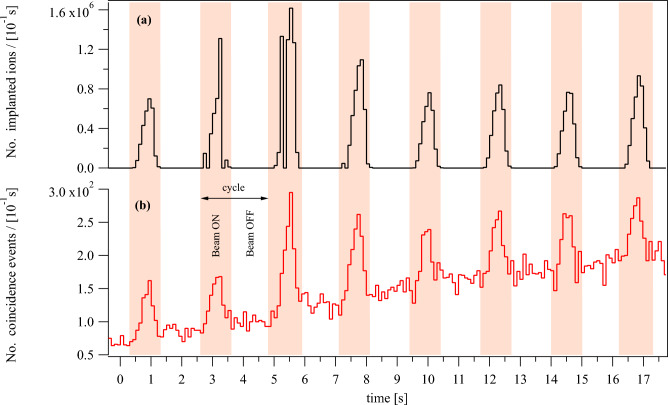
Part of the $^{15}$O low-energy run data as an example of the cycle structure of the secondary beam from the FRS and the time evolution of the coincidence events versus time. The shaded regions mark the periods of beam extraction from the synchrotron and are referred to as “beam ON”. (**a**) The number of implanted ions per 100 ms measured with the ionization chamber and (**b**) the number of recorded coincidence events of positron activity signals per 100 ms measured by the PET scanner. Only coincidence events that occurred during beam-OFF times are used in the analysis.

The time structure of the beam is clearly visible in the intensity measurement by the large area parallel plate ionization chamber (IC) installed in front of the phantom. As an example, the time evolution of the intensity of the $^{15}$O beam as derived from the IC data and the number of coincidence events from the PET data are shown in  Fig. 2. The time structure of the beam is also manifested in the PET data as a higher count rate during beam-on times compared to the beam-off time. The rate increase during beam-ON time can be attributed to the fast-decaying positron-emitting projectile fragments (e.g.$^{9}$C, $^{12}$N, $^{13}$O with half-lives in the millisecond range) produced within the phantom during the stopping process as well as to prompt $\gamma$-emission from excited nuclear levels^39^. These fast-decaying positron-emitters have positrons with high endpoint energies, which broaden the spatial distribution of the positron-emitters and are therefore excluded from the data analysis, see e.g. ^40, 41^.

**Figure 3:**
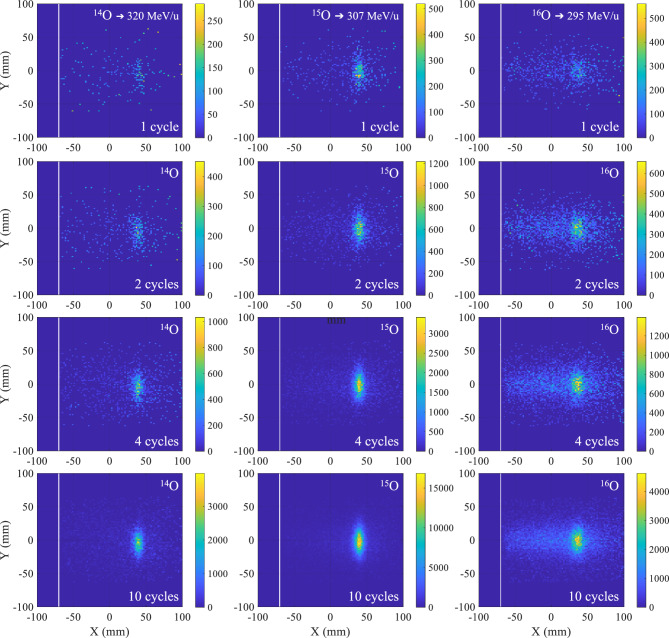
2D PET images obtained during the high-energy implantation of oxygen isotopes in a PMMA phantom. The x and y axes represent the central plane of the beam, which also corresponds to the mid-horizontal plane of the scanner. The beam travels in the positive x-axis direction, and the beam entrance face of the phantom is marked by a white line. The color scale corresponds to the number of coincidence events. Each panel displays the implanted isotope and the total number of implantation cycles prior to the image. The implantation energy of the individual isotopes is provided in the topmost panels. The cycle time structure and beam intensities are given in Table 2. The images have been corrected for PET scanner sensitivity and attenuation in the phantom. The pixel size of the image reconstruction is 2$\times$2 mm$^2$.

**Figure 4:**
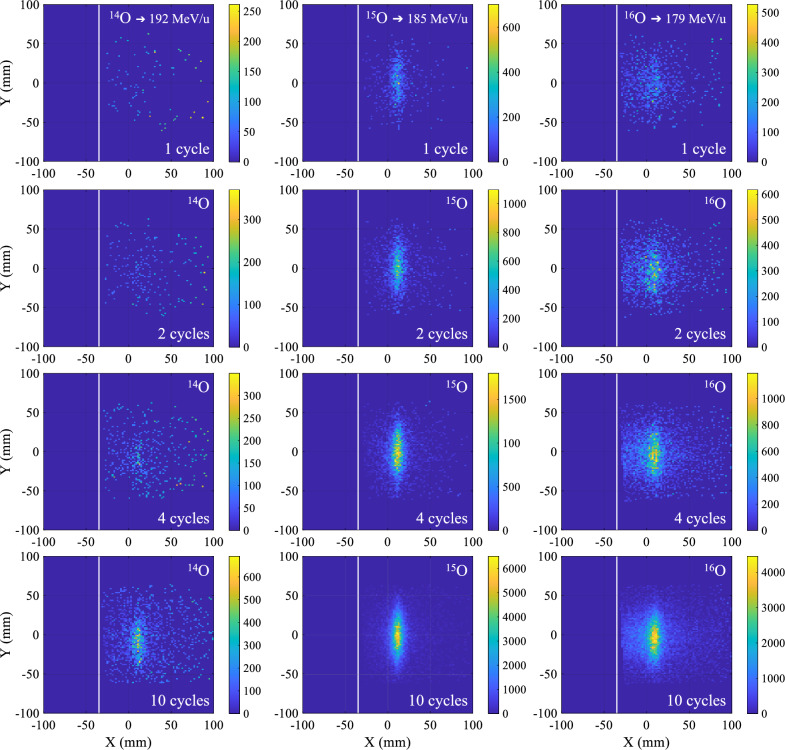
2D PET images obtained during the low-energy implantation of oxygen isotopes in a PMMA phantom. The x and y axes represent the central plane of the beam, which also corresponds to the mid-horizontal plane of the scanner. The beam travels in the positive x-axis direction, and the beam entrance face of the phantom is marked by a white line. The color scale corresponds to the number of coincidence events. Each panel displays the implanted isotope and the total number of implantation cycles prior to the image. The implantation energy of the individual isotopes is provided in the topmost panels. The cycle time structure and beam intensities are given in Table 2. The images have been corrected for PET scanner sensitivity and attenuation in the phantom. The pixel size of the image reconstruction is 2$\times$2 mm$^2$.

 Two detector events, one in each panel, were registered as a coincidence event in event list mode if they occurred within a time window of 4.1 ns and had an energy between 435 and 650 keV. Each coincidence event included a time stamp with 1 ms resolution. The time stamp enabled the monitoring of the time evolution of the positron activity profile during irradiation. The reconstruction procedure used to create 2D PET images of the positron activity profiles is described in detail by Ozoemelam et al.^42^. The 2D image reconstruction of coincidence events was performed using a 2$\times$2 mm$^{2}$ pixel size. Further, the corrections for the attenuation of gamma rays in the phantom and the scanner’s sensitivity must also be taken into account for the accurate quantification of the images. These corrections are specific to the geometrical configuration of the imaging setup. Systematic offline measurements using a $^{22}$Na source were performed to map the sensitivity and attenuation corrections for the two different phantom positions used during the irradiation. A detailed description of the procedure can be found in  Kostyleva et al.^30^ Figures 3 and 4 display the in-beam 2D PET images of $^{14,15,16}$O ions implanted into the PMMA phantom during both the high-energy run and the low-energy run, following various implantation cycles.

#### Fitting of the 1D-positron activity profiles

The 1D-positron activity profiles in the direction of the beam were obtained by laterally integrating the reconstructed 2D images over a region whose width is equal to plus or minus two times the standard deviation of the positron activity distribution.

**Figure 5:**
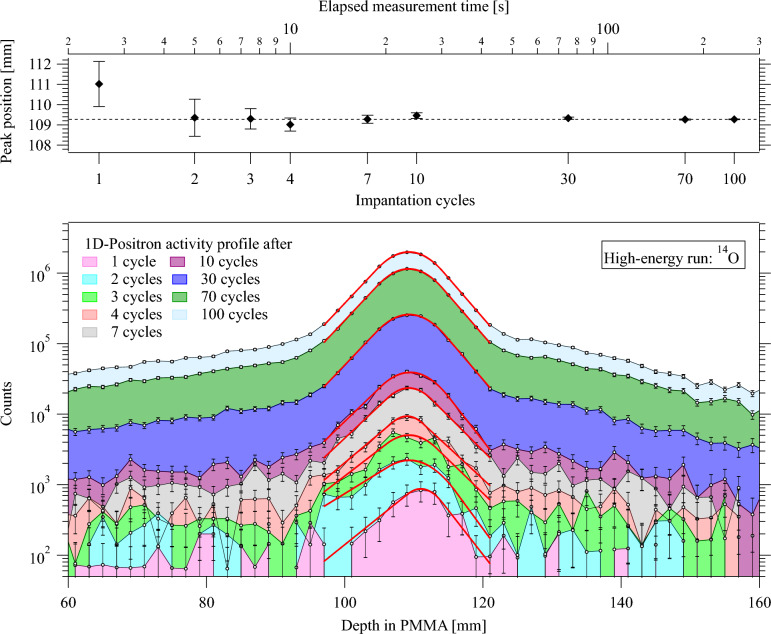
A set of cumulative positron activity profiles from the $^{14}$O high-energy run. Each peak corresponds to the accumulated data after a certain number of cycles. The solid red lines indicate the GE fit to the data. The top panel shows how the peak position evolves over the elapsed measurement time, with the corresponding number of implantation cycles indicated on the bottom axis. The most precise peak position is determined by the measurement with the highest statistics, which is marked as a reference by the dashed line.

To perform further analysis of the 1D-positron activity distributions shown in Fig. 5, an asymmetric peak shape was used to describe the data. Due to its simplicity and the small number of parameters necessary, the so-called GE function, which is a central Gaussian smoothly connected to exponential functions at both or one of the peak edges, was chosen^43^. The capability of the GE function to model the positron activity profiles produced by the implantation of carbon isotopes in a PMMA phantom was successfully demonstrated in the preceding work by Kostyleva et al.^30^ The maximum of the GE peak coincides with the mean of the central Gaussian and the maximum of the positron activity profile is defined by this parameter. It will be referred to as peak position in the following. The data were fitted with the GE function using the curve-fitting routine of the commercial software program Igor Pro. The reduced chi-square ($\chi ^{2}_{red}$) test was used to determine the goodness-of-fit. For the $^{14}$O and $^{15}$O data sets, the GE function represents 90% of the proximal and distal fall-off regions of the positron activity peak. In the case of $^{16}$O, the fit region covers 70% of the proximal and distal fall-off regions. A typical example of a peak analysis procedure to extract the evolution of the peak position over the course of irradiation is demonstrated in Fig. 5 using the high-energy run of $^{14}$O as an example. The precision of range determination is quantified as the standard deviation of the peak position parameter of the GE fitting function. The peak position parameter with the smallest uncertainty is the most precise. It is also considered to be the most accurate value, and it is used as the reference value for the data points with lower statistics. The top panel of Fig. 5 shows the evaluation of the positron activity peak position and its uncertainty over the course of the implantation. Already after the second implantation cycle, the peak position approaches the asymptotic value within the uncertainty and after the third implantation cycle, its uncertainty drops below 1 mm.

## Results

The main focus of this paper is to explore the possibility of fast positron activity range monitoring by quasi-real-time in-beam PET using therapy beams of positron-emitting isotopes of oxygen, $^{15}$O and $^{14}$O. For this purpose, the evaluation of the peak position and its uncertainty during the course of implantation is studied in detail as explained in the previous section.

**Figure 6:**
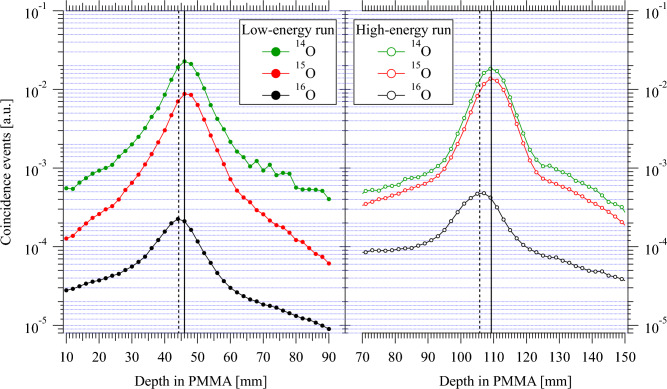
1D positron activity profiles obtained after 100 implantation cycles of $^{14}$O, $^{15}$O and $^{16}$O beams in the PMMA phantom, normalized to the total number of ions implanted during those 100 cycles. Both the low- and high-energy runs are shown. Vertical solid lines indicate the range of the three isotopes calculated with ATIMA 1.2 code using LISE++ program  ^35^. The dashed lines indicate the measured positron activity maxima of $^{16}$O.

### Positron activity profiles of $^{14}$O, $^{15}$O and $^{16}$O beams

Figure 6 shows the 1D positron activity profiles obtained after 100 implantation cycles of $^{14}$O, $^{15}$O and $^{16}$O. The activity profiles are normalized to the total implanted ions. The energy of all three isotopes was adjusted to have the same implantation depth in the phantom. The image peak positions of $^{14}$O, $^{15}$O coincide with each other and their estimated mean range is within the positioning tolerance of the PMMA phantom (±0.25 mm), whereas the positron activity peak of the $^{16}$O has a position proximal to the mean range and is about two orders of magnitude smaller in height.

**Figure 7:**
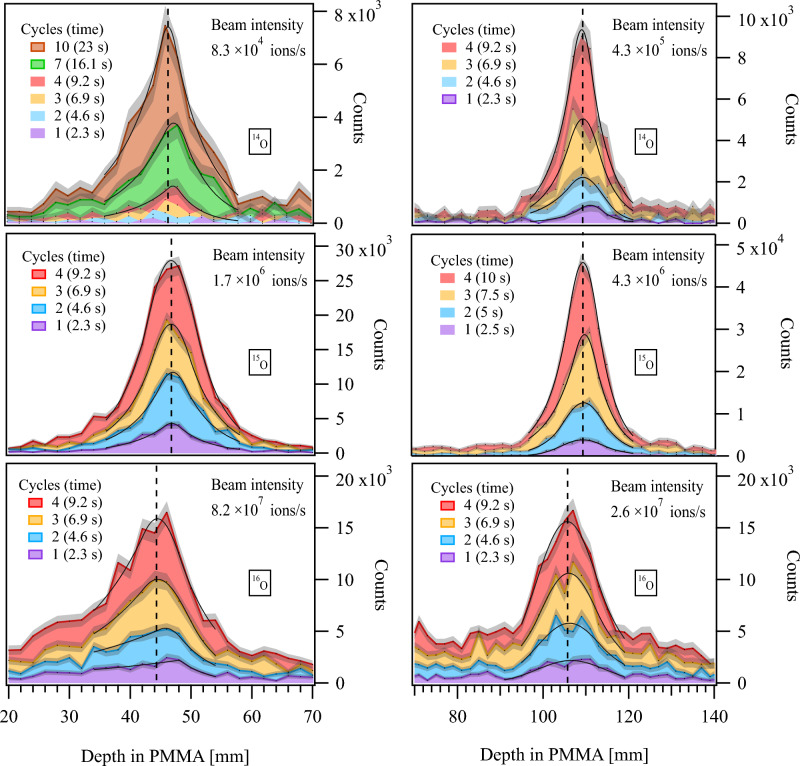
Cumulative 1D positron activity profiles (filled curves) during the first few implantation cycles from both low-energy (left panels) and high-energy runs (right panels) of $^{16}$O, $^{15}$O, and $^{14}$O. The number of cycles is indicated in the legend with the corresponding measurement time given in brackets. The shaded regions represent the associated uncertainties. The solid black lines indicate the GE fit to the data. The average beam intensity during the implantation is shown in the top right corner of each panel. The dashed line indicates the asymptotic value of the peak position.

The positron activity profiles (within the vicinity of the fit region) of $^{16}$O, $^{15}$O, and $^{14}$O over the first few implantation cycles are illustrated in Fig. 7. During this stage, the production cross-sections, half-lives, and ranges of positron-emitting projectile fragments, especially the fast decaying ones like $^{8}$B and $^{10}$C, significantly influence the development of positron activity profiles. As a result, the temporal effect, combined with low statistics, results in significant uncertainty in the fit parameters until the most abundant positron emitter becomes the dominant contributor. For $^{14}$O, the low-energy run yielded no identifiable peak before the fourth implantation cycle. The high-energy run yielded an identifiable peak already on the first implantation cycle due to the fivefold higher intensity (see Table 2). The most significant time-dependent effect on the positron activity profile and peak position is observed in the case of $^{16}$O. Since the primary beam is not a positron-emitter, the positron activity profile is composed of contributions from the positron-emitting projectile fragment peaks superimposed on a plateau formed by the positron-emitting target fragments. In contrast to $^{16}$O, the positron activity peaks resulting from the implantation of $^{15}$O and $^{14}$O are expected to match closely the corresponding ion-beam range. Although significantly less compared to the case of $^{16}$O, the short-lived positron-emitting projectile fragments influence the peak shape until the decays from the projectiles themselves take prominence. In the first few cycles of both the low- and high-energy runs of $^{14}$O and $^{15}$O, the presence of short-lived positron-emitting projectile fragments has a slight but discernible impact on the peak shape. The factor 1.7 shorter half-life of $^{14}$O results in a higher yield of coincidence events compared to that of $^{15}$O for the same number of implanted ions.

Typical uncertainties assumed in robust treatment planning for $^{12}$C ion therapy are a setup uncertainty of ±3 mm and a range uncertainty of ±3.5% of the range^44^. Recent advancements in dual-energy CT technology will allow further reduction of range uncertainty. Regions with greater density changes, such as head and neck tumors, experience higher uncertainties of 2%, whereas, in more homogeneous regions like the liver, uncertainties may be reduced to 1.7%^45^. Range verification should thus have a precision of better than about ±1 mm to have a positive impact on patient treatment.

**Figure 8:**
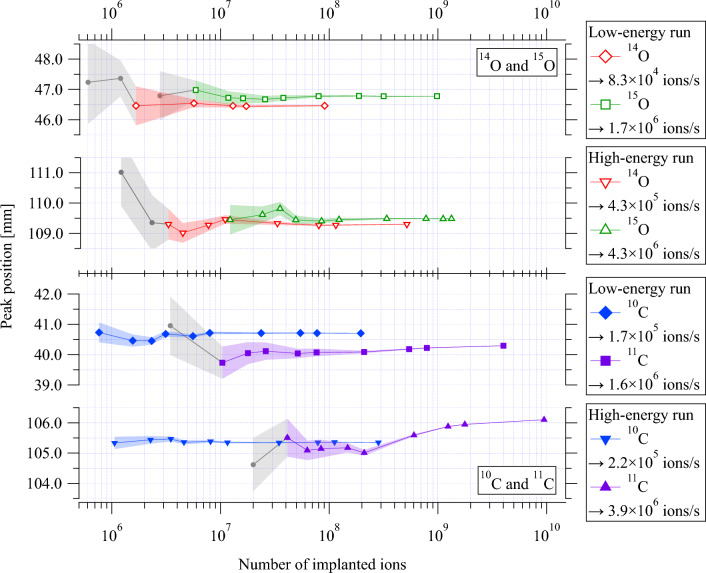
Evolution of the 1D positron activity peak position as a function of the total number of implanted ions, during high and low-energy implantation of $^{15}$O, $^{14}$O (this work) and $^{11}$C, $^{10}$C (from Kostyleva et al.^30^). The average beam intensity used during the implantation is indicated in the legend. The shaded region represents the statistical uncertainties. The drift of the peak position observed in the high-energy run of the $^{11}$C experiment is believed to be caused by the ion-optical instabilities of the $^{12}$C beam entering the FRS section, which can induce slight energy shifts^30^. Data with a deviation of $\ge \pm$0.75 mm from the asymptotic value or with statistical uncertainty $\ge \pm$0.75 mm are indicated by filled gray circles.

In the context of quasi-real-time in-beam PET, the number of implanted ions (which translates to the dose deposition) and the time required for adequate range verification from the start of the implantation are the decisive factors for choosing a candidate. The important questions to be addressed here are:

- What is the minimum number of ions needed to be implanted to determine the positron activity range with a precision that will bring clinical benefit to patients (±1 mm or better)?
- How much time is needed to reach the required precision?

**Figure 9:**
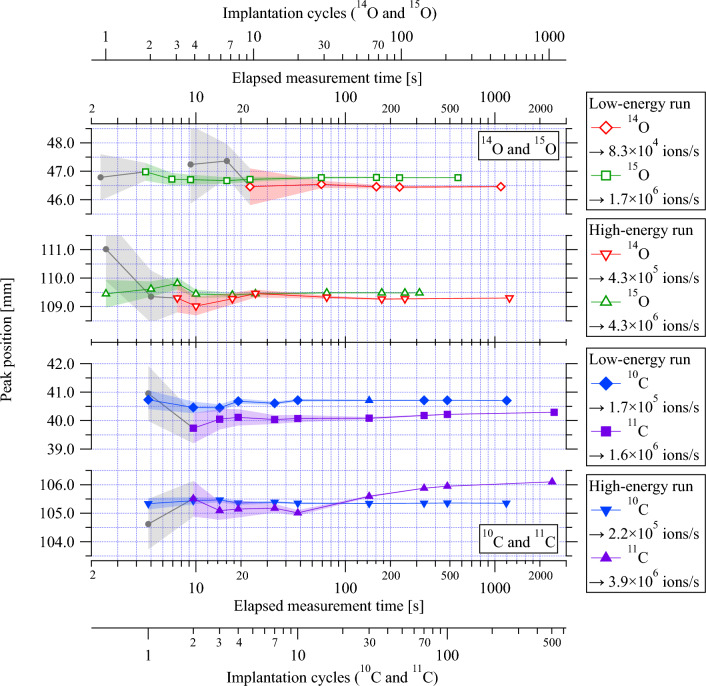
Evolution of the 1D positron activity peak position as a function of elapsed implantation/measurement time, during high- and low-energy implantation of $^{15}$O, $^{14}$O (this work) and $^{11}$C, $^{10}$C (from Kostyleva et al.^30^), the secondary x-axes show the corresponding number of implantation cycles. The average beam intensity used during the implantation is indicated in the legend. The shaded region represents the statistical uncertainties. The drift of the peak position observed in the high-energy run of the $^{11}$C experiment is believed to be caused by the ion-optical instabilities of the $^{12}$C beam entering the FRS section, which can induce slight energy shifts^30^. Data with a deviation of $\ge \pm$0.75 mm from the asymptotic value or with statistical uncertainty $\ge \pm$0.75 mm are indicated by filled gray circles.

To achieve unambiguous range verification by PET in light ion therapy, the detected positron activity distribution must show a distinct peak, and its position and associated uncertainty should be within the expected range of technical (e.g. patient positioning) and biological (i.e., anatomical changes) factors. The conditions for data points to satisfy these criteria are as follows:

- The data point should have an identifiable peak.
- The deviation of the peak position from the asymptotic value (the one obtained from the highest statistics case) should be within $\le \pm$0.75 mm.
- The statistical uncertainty in peak position should be within $\le \pm$0.75 mm.

The value of 0.75 mm is chosen somewhat arbitrarily, but such that it is well within the estimated minimum requirement of ±1 mm. Figures 8 and 9 display the changes in peak position and their uncertainty during the implantation of high- and low-energy $^{15}$O and $^{14}$O ions. These changes are depicted as a function of two variables: accumulated number of implanted ions (Fig. 8) and elapsed measurement/implantation time (Fig. 9). Data from our prior study on positron emitters of carbon, $^{11}$C and $^{10}$C, are included in both figures for comparison ^30^. Compared to Fig. 8, the Fig. 9 provides a clearer picture of the practical aspects of in-beam PET, including the efficiency of producing different isotopes using the in-flight method. The values presented here are specific to the experimental setup’s geometric configuration and reconstruction algorithms used in this experiment, as detailed in Kostyleva et al.’s work^30^. It should be noted that  the results can be easily scaled to other PET systems.

## Discussion

PET scanners can determine the range of positron activity with a certain precision by collecting a certain minimum number of counts. The actual number of counts required depends on the geometry and sensitivity of the scanners. From an instrumental perspective, the slower decay rate of long-lived positron emitters compared to short-lived ones can be compensated by higher intensity. However, for therapeutic applications, the required dose, and consequently, the number of ions implanted is defined by medical requirements. Therefore, it is advantageous to achieve range verification as quickly as possible with the smallest possible dose.

Figure 9 compares positron emitters in quasi-real-time in-beam PET, illustrating the impact of half-life and intensity on image quality. It also helps to infer the influence of implantation cycle time structure on coincidence events per cycle. For instance, in the high-energy run, $^{10}$C shows better precision than $^{15}$O by the end of the first beam cycle, despite its 20 times lower beam intensity. This result is due to $^{10}$C’s shorter half-life, 6 times less than $^{15}$O, with two-fold longer implantation (2.0 seconds beam-ON) and decay (2.8 s beam-OFF) times. A quantitative comparison is challenging due to coupled factors like half-life, beam intensity, and different cycle time structures used for oxygen and carbon experiments. To facilitate comparison, a formulation considering these factors is essential. In this experiment, positron-emitting secondary beams were generated via projectile fragmentation of primary beams ($^{12}$C and $^{16}$O ions). The secondary beam intensity ($I_{s}$) in ions/pulse is given by:

1 $$\begin{aligned} I_{s} =\eta I_{0}, \end{aligned}$$

where $\eta$ is the conversion factor (see Table 3), representing the ratio of positron-emitting ions delivered to the imaging phantom ($I_{s}$) to primary ions accelerated in the synchrotron ($I_{0}$). $\eta$ is given by:

2 $$\begin{aligned} \eta = \sigma _{cs}\rho _{A}\epsilon , \end{aligned}$$

where $\sigma _{cs}$ is the production cross-section (see Table 3), and $\rho _{A}$ is the areal density of the production target (in number of atoms/cm$^{2}$). $\epsilon$ is an efficiency factor defined as the ratio of the isotope beam intensity available for imaging to the intensity of the reaction products produced in the primary reaction. Irradiation is performed in a pulsed mode with a beam-ON time $t_p$ and a subsequent beam-OFF time $t_r$. The beam cycle time *T* is defined as $t_p+t_r$. The number of positron emitters $N_{0}$ accumulated by the end of a beam-ON time is given by:

3 $$\begin{aligned} N_{0}=\frac{I_{s}}{\lambda t_p}\left[ 1-e^{-\lambda t_{p}}\right] =\frac{\eta I_{0}}{\lambda t_p}\left[ 1-e^{-\lambda t_{p}}\right] , \end{aligned}$$

where $\lambda =\ln (2)/t_{1/2}$ is the decay constant. The number of decays during the subsequent beam-OFF time $t_r$, $N_{D}$, is given by:

4 $$\begin{aligned} N_{D}=N_{0}-N_{0}e^{-\lambda t_{r}}=\frac{\eta I_{0}}{\lambda t_p}\left[ 1-e^{-\lambda t_{p}}\right] \left[ 1-e^{-\lambda (T-t_{p})}\right] . \end{aligned}$$

**Table 3:** The primary beams, their intensities, the conversion factors, of the oxygen and carbon positron emitters produced by FRS for the high-energy implantation.

Primary beam	Primary beam intensity	Conversion factor	Production cross-section^46^	Secondary beam
	$I_{0}$ [ions/pulse]	$\eta$	$\sigma _{cs}$ [10$^{-24}$ cm$^{2}$]	
$^{16}$O	3$\times$10$^{9}$	4.3$\times$10$^{-3}$	43	$^{15}$O
	9$\times$10$^{9}$	1.2$\times$10$^{-4}$	1.2	$^{14}$O
$^{12}$C	8$\times$10$^{9}$	2.4$\times$10$^{-3}$	46.7	$^{11}$C
	1$\times$10$^{10}$	1.1$\times$10$^{-4}$	4.3	$^{10}$C

The production cross sections are experimentally obtained values from Lindstrom et al.^46^.

**Figure 10:**
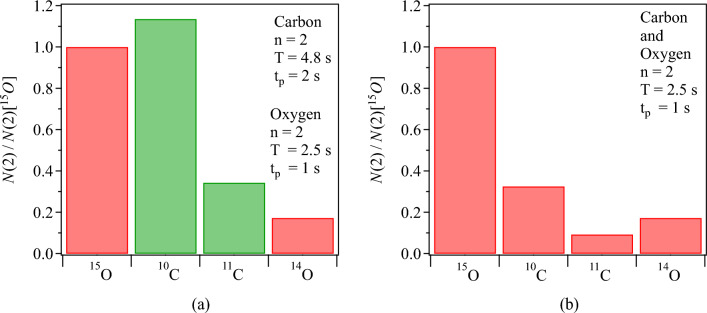
Calculated yields of coincidence events of $^{15}$O, $^{10}$C, $^{11}$C and $^{14}$O normalized to the yield of $^{15}$O using Eq. 5 with n=2. The values for the conversion factor $\eta$ and primary beam intensity $I_{0}$ in Eq. 5 are the experimental ones as listed in Table 3. The cycle structure used in panel (**a**) is $T= 2.5~s$, $t_{p}=1~s$ for oxygen positron emitters and $T= 4.8~s$, $t_{p}=2~s$ for carbon positron emitters as it is the experimental data from the high energy runs presented in Fig. 9. Panel (**b**) shows the expected relative yields if all isotopes share the same cycle time structure, here shown for $T=2.5~s$, $t_{p}=1~s$.

Equation 4 represents the number of decays, and therefore also the number of coincidence events recorded during the beam-OFF time. This is because only these coincidence events are used for image reconstruction and further analysis, see Fig. 2. This equation is applicable for the first beam cycle. For subsequent pulses, it becomes essential to consider the contribution of decay from positron emitters deposited in preceding implantation cycles. The total number of decays accumulated after after *n* implantation cycles is thus given by:

5 $$\begin{aligned} N(n)=N_{D}\sum _{j=0}^{(n-1)}(n-j)e^{-\lambda j T}. \end{aligned}$$

The calculated relative yield of coincidence events, as depicted in Fig. 10a, suggests that the model presented in Eq. 5 effectively captures the data showcased in Fig. 9. Notably, the 13% higher coincidence yield of $^{10}$C compared to $^{15}$O can be attributed to the favorable beam cycle time structure of the carbon experiment. To facilitate a fair comparison of the performance among different positron emitters it is crucial to eliminate the influence of the difference in the beam cycle time between the oxygen and carbon experiments. To achieve this, the relative yield of coincidence events for various positron emitters is calculated under the assumption that they share the same beam cycle time structure, and is presented in Fig. 10b. The beam cycle time structure from the oxygen experiment was employed for these calculations. This analysis reveals that if the beam cycle time structure is standardized across all isotopes presented here, $^{15}$O emerges as the superior candidate.

**Figure 11:**
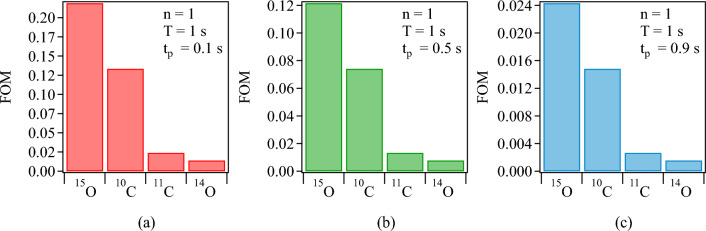
Calculated Figure of Merit (FOM) for the first beam cycle of therapy-relevant positron-emitting beams, $^{15}$O, $^{10}$C, $^{11}$C, and $^{14}$O, for a cycle time $T=1$s and three different beam-ON times: (**a**) $t_p=0.1$s, (**b**) $t_p=0.5$s, and (**c**) $t_p=0.9$s.

In Eq. 5, the primary beam intensity $I_{0}$ and the components of the conversion factor $\eta$, namely the areal density of the target $\rho _{A}$ and the efficiency of beam transport $\epsilon$, are system-dependent parameters (see Eqs. 1 and 2). To extend the results of this work to a system-independent context, it is advantageous to introduce a Figure of Merit (FOM) that is based on two decisive fundamental quantities: the half-life and the production cross-section. The production cross-section addresses the availability of therapy-relevant intensities, while the half-life of the positron-emitter, along with the beam cycle structure, addresses the quasi-real-time PET feedback aspects of the positron emitters under consideration.

To construct the FOM, it is assumed that all system-dependent factors, $I_{0}$, $\rho _{A}$, and $\epsilon$, are approximately the same for all isotopes, and their values are set to unity; i.e., $I_{0}$ is equal to 1 ion/pulse, $\rho _{A}$ is equal to 1 atom/cm$^{2}$, and $\epsilon$ is equal to 1. The resulting FOM is a dimensionless value that is proportional to *N*(*n*) (see Eq. 5).

To illustrate the application of the Figure of Merit (FOM), results for $^{15}$O, $^{14}$O, $^{11}$C, and $^{10}$C are presented in Fig. 11 for the first beam cycle for the same cycle time *T* but three different beam-ON times $t_p$. These examples show that, regardless of the beam cycle time structure, $^{15}$O consistently emerges as the superior candidate, confirming the experimental results obtained in this work.

In terms of the half-live, $^{10}$C is the best candidate for quasi-real-time range monitoring. However, the production cross-section of $^{10}$C is an order of magnitude lower than that of $^{11}$C and $^{15}$O. This is also true for $^{14}$O. This implies that the production of $^{10}$C and $^{14}$O requires an order of magnitude higher intensity from the driver accelerator to reach therapeutical intensities. In the case of $^{11}$C, the half-life of 1221.8 s makes it not optimal for quasi-real-time range monitoring using in-beam PET. For the therapy-relevant positron emitter $^{13}$N, the same holds true^47^. From measurements (see Figs. 9 and 10b) $^{15}$O comes out as a clear favorite among all considered isotopes for quasi-real-time range monitoring by in-beam PET during therapy as quantified in the figure of merit analysis. It must be noted that the characteristic fragmentation tail of heavy ions is higher for oxygen compared to carbon. This issue is unavoidable but relevant in the choice of therapeutic isotope. A comprehensive discussion of this matter is beyond the scope of this work.

A systematic investigation was conducted on the production of $^{11}$C and $^{15}$O using the in-flight method at HIMAC, Japan. The study demonstrated the feasibility of producing $^{15}$O beams suitable for medical treatment, with a reported production yield of 0.43(2)% and 97.2% purity, achieved through projectile fragmentation of a 430 MeV/u $^{16}$O beam. Similarly, a production yield of 0.76% with 99% purity was reported for $^{11}$C by projectile fragmentation of a 430 MeV/u $^{12}$C beam at HIMAC, the maximum primary beam intensities available for $^{16}$O and $^{12}$C are 1.1$\times$10$^{9}$ ions/s and 1.9$\times$10$^{9}$ ions/s, respectively^19, 21^. The present study also demonstrates the feasibility of producing $^{15}$O beams suitable for medical treatment using an in-flight method, achieving, purity, and energies required for the purpose (see Table 2).

With the method and parameters used in this work (including the intensity of the primary beam, energy of the primary and secondary beam, production target material and thickness, phase space acceptance of the separator, choice of degrader material and thickness), the beam intensity of $^{15}$O exceeds that of $^{14}$O by a factor of 10 to 20. The conversion factors for the high-energy runs of $^{15}$O and $^{14}$O are provided in Table 3, while for the low-energy runs, they are 8$\times$10$^{-5}$ for $^{14}$O and 1$\times$10$^{-4}$ for $^{15}$O. The higher conversion factor of $^{15}$O compared to $^{14}$O results primarily from its greater production cross-section, while the disparity between low and high energy primarily arises from differences in transmission through the separator^48^. The conversion factors for analogous studies conducted with carbon isotopes are presented in Table 3. These beams have a similar implantation depth as the high-energy run of oxygen. The two times higher conversion factor for $^{15}$O compared to $^{11}$C is because the smaller emittance of the former leads to a higher transmission through the FRS spectrometer.

It should be noted that the maximum intensity achieved for $^{16}$O in the SIS18 is 10$^{11}$ ions/cycle^49^. Therefore, there is potential to increase the intensity of $^{15}$O at the symmetric branch of FRS by an order of magnitude. This would make it comparable to the typical intensity of about 5$\times$10$^{8}$ ions/s available at a $^{12}$C treatment facility^50^.

ISotope On-Line (ISOL) is a method alternative to in-flight for RIB production. CRC Louvain-la-Neuve in Belgium has produced a low-energy beam ISOL beam of $^{15}$O with an intensity of 6$\times$10$^{7}$ ions/s, and LBNL has produced a $^{14}$O beam with an intensity of 3$\times$10$^{7}$ ions/s^51–53^. The highest intensity achieved for an ISOL beam of $^{11}$C is 1$\times$10$^{8}$ ions/s by LBNL^54^. Although the ISOL technique produces highly pure and intense Radioactive Ion Beams (RIB), the production of a RIB with sufficient energy for ion beam therapy using the ISOL technique has not yet been demonstrated. A recent Technical Design Report (TDR)^50^ published by the MEDICIS-Promed network^55^ provides a comprehensive exploration of implementing ISOL-produced post-accelerated $^{11}$C radioisotopes in particle therapy centers. The TDR presents findings from a thorough review of clinical applicability, utilizing a combination of experimental data and Monte Carlo simulations. This in-depth study covers various technical solutions, including production, ionization, acceleration, and transport, with a specific emphasis on upgrading existing $^{12}$C particle therapy centers. These findings can be easily extended to therapy beams involving $^{15}$O.

The emergence of high-LET FLASH radiotherapy^56^ as a potential breakthrough in cancer treatment has sparked interest in optimizing its delivery since it is characterized by the delivery of an ultra-high dose of radiation in a single pulse. By incorporating in-beam quasi-real-time range verification offered by the $^{15}$O beam, the precision and safety of high-LET FLASH therapy can be significantly enhanced. An ion beam therapy center equipped with a $^{15}$O beam presents a unique and promising opportunity to perform in-beam range verification using a low-intensity probe pulse prior to the FLASH.

## Conclusions

This study provides a comprehensive quantitative and qualitative comparison of the therapy-relevant beams of positron-emitting isotopes of carbon and oxygen within the context of quasi-real-time range verification capability. $^{11}$C is currently the most researched candidate for therapeutic RIB due to its enhanced imaging potential without dosimetric drawbacks, but its half-life is too long to be considered for fast range monitoring using in-beam PET. The lower production cross-section of $^{10}$C and $^{14}$O makes it challenging to produce them with intensities of therapeutical needs. The results also demonstrate that, from the perspective of an in-flight production and separation method, $^{15}$O is the better choice in terms of achievable intensity. In conclusion, $^{15}$O is the most technically feasible choice for a therapeutic beam that allows quasi-real-time range monitoring by in-beam PET due to its faster response at a lower dose. This study also demonstrates the feasibility of producing $^{15}$O beams with an intensity, purity, and energy suitable for ion-beam therapy using the method of $^{16}$O-projectile fragmentation and separation in-flight.

Further research and development in this field have the potential to significantly advance ion beam therapy techniques and improve treatment outcomes for cancer patients. For example, by incorporating in-beam quasi-real-time range verification and utilizing the positron-emitting oxygen beam, the precision and safety of high-LET FLASH therapy can be substantially enhanced.

## Data Availability

The datasets used and/or analysed during the current study can be made available under a collaboration agreement with the GSI Helmholtzzentrum für Schwerionenforschung GmbH, Darmstadt, Germany. Please contact the corresponding authors for more information.

## References

[1] Durante M, Debus J, Loeffler JS (2021). Physics and biomedical challenges of cancer therapy with accelerated heavy ions. Nat. Rev. Phys..

[2] Durante M, Orecchia R, Loeffler JS (2017). Charged-particle therapy in cancer: Clinical uses and future perspectives. Nat. Rev. Clin. Oncol..

[3] Kurz C, Mairani A, Parodi K (2012). First experimental-based characterization of oxygen ion beam depth dose distributions at the Heidelberg ion-beam therapy center. Phys. Med. Biol..

[4] Habermehl D (2014). The relative biological effectiveness for carbon and oxygen ion beams using the raster-scanning technique in hepatocellular carcinoma cell lines. PLoS ONE.

[5] Tommasino F, Scifoni E, Durante M (2015). New ions for therapy. Int. J. Part. Ther..

[6] Dokic I (2016). Next generation multi-scale biophysical characterization of high precision cancer particle radiotherapy using clinical proton, helium-, carbon-and oxygen ion beams. Oncotarget.

[7] Sokol O (2017). Oxygen beams for therapy: Advanced biological treatment planning and experimental verification. Phys. Med. Biol..

[8] Tinganelli W (2015). Kill-painting of hypoxic tumours in charged particle therapy. Sci. Rep..

[9] Ebner DK, Frank SJ, Inaniwa T, Yamada S, Shirai T (2021). The emerging potential of multi-ion radiotherapy. Front. Oncol..

[10] Sokol O (2022). Potential benefits of using radioactive ion beams for range margin reduction in carbon ion therapy. Sci. Rep..

[11] Parodi K (2021). Ion range and dose monitoring with positron emission tomography. Radiation Therapy Dosimetry: A Practical Handbook.

[12] Parodi K (2012). PET monitoring of hadrontherapy. Nucl. Med. Rev..

[13] Fiedler F, Kunath D, Priegnitz M, Enghardt W (2012). Online irradiation control by means of PET. Ion Beam Therapy.

[14] Handrack J (2017). Sensitivity of post treatment positron emission tomography/computed tomography to detect inter-fractional range variations in scanned ion beam therapy. Acta Oncol..

[15] Nischwitz SP (2015). Clinical implementation and range evaluation of in vivo pet dosimetry for particle irradiation in patients with primary glioma. Radiother. Oncol..

[16] Sommerer F (2009). In-beam pet monitoring of mono-energetic 16O and 12C beams: Experiments and FLUKA simulations for homogeneous targets. Phys. Med. Biol..

[17] Kraan AC (2015). Range verification methods in particle therapy: Underlying physics and Monte Carlo modeling. Front. Oncol..

[18] Castro JR (1980). Current status of clinical particle radiotherapy at Lawrence Berkeley laboratory. Cancer.

[19] Kanazawa M (2002). Application of an RI-beam for cancer therapy: In-vivo verification of the ion-beam range by means of positron imaging. Nucl. Phys. A.

[20] Durante M, Parodi K (2020). Radioactive beams in particle therapy: Past, present, and future. Front. Phys..

[21] Mohammadi A (2017). Production of an $$^{15}$$O beam using a stable oxygen ion beam for in-beam PET imaging. Nucl. Instrum. Methods Phys. Res. Sect. A.

[22] Mohammadi A (2019). Range verification of radioactive ion beams of $$^{11}$$C and $$^{15}$$O using in-beam PET imaging. Phys. Med. Biol..

[23] Chacon A (2020). Experimental investigation of the characteristics of radioactive beams for heavy ion therapy. Med. Phys..

[24] Mizuno H (2003). Washout measurement of radioisotope implanted by radioactive beams in the rabbit. Phys. Med. Biol..

[25] Tomitani T (2003). Washout studies of 11C in rabbit thigh muscle implanted by secondary beams of HIMAC. Phys. Med. Biol..

[26] Toramatsu C (2018). Washout effect in rabbit brain: In-beam PET measurements using $$^{10}$$C, $$^{11}$$C and $$^{15}$$O ion beams. Biomed. Phys. Eng. Express.

[27] Toramatsu C (2022). Measurement of biological washout rates depending on tumor vascular status in 15O in-beam rat-pet. Phys. Med. Biol..

[28] Boscolo D (2021). Radioactive beams for image-guided particle therapy: The BARB experiment at GSI. Front. Oncol..

[29] Boscolo D (2022). Depth dose measurements in water for 11C and 10C beams with therapy relevant energies. Nucl. Instrum. Methods Phys. Res. Sect. A Accel. Spectrom. Detect. Assoc. Equip..

[30] Kostyleva D (2022). Precision of the PET activity range during irradiation with 10C, 11C, and 12C beams. Phys. Med. Biol..

[31] Haettner E (2023). Production and separation of positron emitters for hadron therapy at FRS-Cave M. Nucl. Instrum. Methods Phys. Res. Sect. B.

[32] Levin CS, Hoffman EJ (1999). Calculation of positron range and its effect on the fundamental limit of positron emission tomography system spatial resolution. Phys. Med. Biol..

[33] Blumenfeld Y, Nilsson T, Van Duppen P (2013). Facilities and methods for radioactive ion beam production. Phys. Scr..

[34] Geissel H (1992). The GSI projectile fragment separator (FRS): A versatile magnetic system for relativistic heavy ions. Nucl. Instrum. Methods Phys. Res. Sect. B.

[35] Tarasov O, Bazin D (2008). LISE++: Radioactive beam production with in-flight separators. Nucl. Instrum. Methods Phys. Res. Sect. B Beam Interact. Mater. Atoms.

[36] Stelzer, H. & Voss, B. Ionization chamber for ion beams and method for monitoring the intensity of an ion beam (2002). U.S. Patent No. 6,437,513.

[37] Jakoby B (2011). Physical and clinical performance of the mCT time-of-flight PET/CT scanner. Phys. Med. Biol..

[38] Gotoh H, Yagi H (1971). Solid angle subtended by a rectangular slit. Nucl. Inst. Methods.

[39] Parodi K (2005). Random coincidences during in-beam pet measurements at microbunched therapeutic ion beams. Nucl. Instrum. Methods Phys. Res. Sect. A.

[40] Pawelke J (1997). In-beam PET imaging for the control of heavy-ion tumour therapy. IEEE Trans. Nucl. Sci..

[41] Parodi K, Enghardt W, Haberer T (2002). In-beam PET measurements of $$\beta ^+$$ radioactivity induced by proton beams. Phys. Med. Biol..

[42] Ozoemelam I (2020). Feasibility of quasi-prompt PET-based range verification in proton therapy. Phys. Med. Biol..

[43] Routti J, Prussin S (1969). Photopeak method for the computer analysis of gamma-ray spectra from semiconductor detectors. Nucl. Inst. Methods.

[44] Wolf M, Anderle K, Durante M, Graeff C (2020). Robust treatment planning with 4D intensity modulated carbon ion therapy for multiple targets in stage IV non-small cell lung cancer. Phys. Med. Biol..

[45] Peters N (2022). Reduction of clinical safety margins in proton therapy enabled by the clinical implementation of dual-energy CT for direct stopping-power prediction. Radiother. Oncol..

[46] Lindstrom, P., Greiner, D., Heckman, H., Cork, B. & Beiser, F. Isotope production cross sections from the fragmentation of 16O and 12C at relativistic energies (1975). Lawrence Berkeley National Laboratory Report No.:LBL-3650, https://escholarship.org/uc/item/1ff1c88f.

[47] Cassidy D, Canter K, Shefer R, Klinkowstein R, Hughey B (2002). Positron beam production with a deuteron accelerator. Nucl. Instrum. Methods Phys. Res. Sect. B.

[48] Geissel H (1989). Ions penetrating through ion-optical systems and matter-non-liouvillian phase-space modelling. Nucl. Instrum. Methods Phys. Res., Sect. A.

[49] Spiller, P. *et al.* SIS18 status report. GSI Sci. Rep. (2011).

[50] Penescu L (2022). Technical design report for a carbon-11 treatment facility. Front. Med..

[51] Loiselet M (2001). The production and generation of radioactive beams at louvain-la-neuve. AIP Conf. Proc..

[52] Burke J (2002). Development of a low-energy oxygen 14 ion beam. Rev. Sci. Instrum..

[53] Köster U (2002). Intense radioactive-ion beams produced with the ISOL method. Eur. Phys. J. A.

[54] Powell J (2000). BEARS: Radioactive ion beams at Berkeley. Nucl. Instrum. Methods Phys. Res. Sect. A.

[55] Cocolios, T. E., Ferrari, C., Reid, F. & Stora, T. Medicis-promed: An innovative training network for a new generation of professionals in nuclear medicine. In *Proc. EMBEC & NBC 2017: joint conference of the european medical and biological engineering conference (EMBEC) and the nordic-baltic conference on biomedical engineering and medical physics (NBC)* 530–533 (Tampere, Finland, 2017), 10.1007/978-981-10-5122-7_133 (Springer, 2018).

[56] Weber UA, Scifoni E, Durante M (2022). Flash radiotherapy with carbon ion beams. Med. Phys..

